# Anthropology

**Published:** 1907-06-01

**Authors:** 


### Anthropology.

The announcement that the Cambridge Board of Anthropological Studies has recommended, in a special report to the Senate, the establishment of a Diploma in Anthropology, will be received with gratification by all friends of the University, and with equal satisfaction by those who are interested in the extension and development of higher education. The neglect of the study of anthropology, as a science and not merely as an absorbing hobby, at most of our universities is a matter for regret, and this step of recognising the study as one that should have its place in the scientific curriculum will meet with the approval of all thoughtful men. There is little need, in a medical paper and to medical readers, to emphasise or lay stress upon the importance of the study of what Virchow, himself one of the foremost anthropologists of his day, called "the most fascinating of sciences." The practical advantages such study offers may, it is true, be few. The scientific anthropologist may find it impossible to make a living by his knowledge of the science: and that, in our days when most studies are valued at their commercial price, may in itself be a drawback. But to those of us who agree with Milton that "not liberal indeed but illiberal must be the mind that mounts in contemplation merely for money," anthropology must of necessity offer many points of interest, of value, and of solid usefulness.

The proper study of mankind, as Lucretius pointed out and Pope after him, is man himself, his race, his development, and his reaction to environment. In its best, its most liberal interpretation, that is the meaning which may be assigned to the word "Anthropology." It is not a mere study of the cranial development of aboriginal races, an anthropometric survey of any particular tribe, or a statistical enumeration of ethnological data. It is the sympathetic, broad-minded, and, at the same time, scientific study of the human race in all its varied aspects. As such it appeals particularly to the medical man and the medical student. Only by correlating and carefully sifting all the available data can we arrive at a proper knowledge of certain outstanding questions of, for instance, racial susceptibility to disease, the evolution of mind and the origin of social conditions. Excellent work has been done in the past by missionaries, by enthusiastic general practitioners abroad, and by equally enthusiastic experts at home, but such work has, to a great extent, been sporadic, and has hitherto failed to receive the official recognition that it merited.

Most of our larger museums contain anthropological and ethnographical collections which will be of undoubted value to the student who desires to avail himself of the facilities which, it appears, Cambridge, itself the fortunate possessor of one of the finest anthropological collections in the kingdom, offers to the intending Diplomate in Anthropology. Here and there, too, workers are adding to the known data, and increasing the facilities for definite study and original research. At University College, for example, Professor Karl Pearson's admirable work in the department of National Eugenics has already borne fruit, though much, very much, remains to be added to it before we are in a position to estimate it at its proper worth. On the Continent various workers have been active on similar, or analogous lines, and he who would give us a survey of what has been accomplished so far, collating the findings of these various investigators, will do a work for the accomplishment of which scientific men will feel cordially grateful.

So far as the report of the Board goes, it would appear that the aspirant for the Diploma in Anthropology will not be confined to one subject, nor will his researches be limited to one branch of a study which is so complex and so wide ranging. He is to be left free to pursue his own bent, and to strike out in any line he wishes. That is a decision which is eminently praiseworthy. To have given the candidate set work, to have limited his choice of investigation, or to have narrowed his field of activity, would have been to stultify much of the good intention of the recommendation, which is, as we take it, to encourage original work by recognising the manysidedness of the study to which the aspiring diplomate will have to devote himself.

